# Bicarbonate Induced Redox Proteome Changes in Arabidopsis Suspension Cells

**DOI:** 10.3389/fpls.2017.00058

**Published:** 2017-01-26

**Authors:** Zepeng Yin, Kelly Balmant, Sisi Geng, Ning Zhu, Tong Zhang, Craig Dufresne, Shaojun Dai, Sixue Chen

**Affiliations:** ^1^Plant Molecular and Cellular Biology Program, Department of Biology, Genetics Institute, University of FloridaGainesville, FL, USA; ^2^Key Laboratory of Saline-alkali Vegetation Ecology Restoration in Oil Field, Alkali Soil Natural Environmental Science Center, Ministry of Education, Northeast Forestry UniversityHarbin, China; ^3^Interdisciplinary Center for Biotechnology Research, University of FloridaGainesville, FL, USA; ^4^Thermo Fisher ScientificWest Palm Beach, FL, USA

**Keywords:** *Arabidopsis thaliana*, bicarbonate, CO_2_, iTRAQ, iodoTMT, redox proteomics, oxidative stress

## Abstract

Climate change as a result of increasing atmospheric CO_2_ affects plant growth and productivity. CO_2_ is not only a carbon donor for photosynthesis but also an environmental signal that can perturb cellular redox homeostasis and lead to modifications of redox-sensitive proteins. Although redox regulation of protein functions has emerged as an important mechanism in several biological processes, protein redox modifications and how they function in plant CO_2_ response remain unclear. Here a new iodoTMTRAQ proteomics technology was employed to analyze changes in protein redox modifications in *Arabidopsis thaliana* suspension cells in response to bicarbonate (mimic of elevated CO_2_) in a time-course study. A total of 47 potential redox-regulated proteins were identified with functions in carbohydrate and energy metabolism, transport, ROS scavenging, cell structure modulation and protein turnover. This inventory of previously unknown redox responsive proteins in Arabidopsis bicarbonate responses lays a foundation for future research toward understanding the molecular mechanisms underlying plant CO_2_ responses.

## Introduction

The global atmospheric concentration of carbon dioxide (CO_2_) has increased from 280 μmol mol^−1^ during the pre-industrial period to 388.5 μmol mol^−1^ in 2010 (NOAA/ESRL, http://www.esrl.noaa.gov/gmd/ccgg/trends/) and is projected to increase to 700 μmol mol^−1^ by the end of the twenty first century (Prentice et al., 2001; Aranjuelo et al., 2011). CO_2_ is not only a carbon donor for photosynthesis but also an environmental signal that regulates stomatal movement and thereby controls water transpiration and carbon fixation (Tian et al., 2015). The influence of increasing atmospheric CO_2_ on plant physiology (photosynthesis, respiration, and stomatal conductance) has been studied in different species (Urban, 2003; Ainsworth and Long, 2005). However, proteomic level changes under elevated CO_2_ conditions have not been studied in single cell types.

A small fraction of CO_2_ can react with H_2_O to form bicarbonate (Xue et al., 2011). This interconversion between CO_2_ and bicarbonate can be accelerated by carbonic anhydrases (CAs), which favor the formation of bicarbonate under normal plant growth conditions (Badger, 1994). Hence, bicarbonate can be used to mimic the effects of elevated CO_2_ (Kolla et al., 2007). Reactive oxygen species (ROS) are important players in many signaling and metabolic pathways (Baxter et al., 2014), including elevated CO_2_ signaling in wheat (Rao et al., 1995) and *Ginkgo biloba* (Lu et al., 2009). In general, low concentrations of ROS function in signal transduction leading to activation of defense responses (Mittler et al., 2004), while high levels lead to oxidative damage of lipids, DNA, and proteins (Ghezzi and Bonetto, 2003). Therefore, the balance between ROS production and ROS scavenging is crucial, and ROS detoxification depends on various ROS scavenging enzymes and antioxidants (e.g., ascorbate (AsA) and glutathione (GSH); Mittova et al., 2004; Miller et al., 2010). Previous studies showed that elevated CO_2_ decreased SOD activities in spruce, pine and oak (Polle et al., 1993; Schwanz et al., 1996a,b) and reduced catalase (CAT) activities in spruce and tobacco (Havir and McHale, 1989; Polle et al., 1993). The result could be explained by a high CO_2_:O_2_ ratio within chloroplasts, which would decrease electron leakage from PSI to O_2_, thereby attenuating ${\text{O}}_{2}^{-}$ formation (Schwanz et al., 1996b). Moreover, the increased ratio of CO_2_:O_2_ would decrease the oxygenase activity of Rubisco in favor of carboxylation, thus reducing photorespiration and resultant cellular H_2_O_2_ production, and therefore low oxidative damage and demand for ROS detoxification (Qiu et al., 2008). In contrast, some other experiments showed that elevated CO_2_ may result in oxidative stress to cause increased activities of antioxidant enzymes (Rao et al., 1995; Marabottini et al., 2001; Schwanz and Polle, 2001). Because of these contrasting results about the effect of elevated CO_2_ on redox enzyme activities, here we conducted detailed analyses of redox enzymes using *A. thaliana* suspension cells.

To improve understanding of elevated CO_2_ responses, transcriptomic analyses have been carried out in several species, which include *A. thaliana* (Li et al., 2008; Kanani et al., 2010; Kaplan et al., 2012), *Oryza sativa* (Fukayama et al., 2011), *Populus euramericana* (Tallis et al., 2010), *Zea mays* (Prins et al., 2011), and *Triticum aestivum* (Nie et al., 1995). The transcriptomics studies showed that elevated CO_2_ caused significant changes in many metabolic pathways and physiological processes, e.g., cellular redox homeostasis and carbohydrate metabolism. Previous metabolomic analyses in several species showed that the CO_2_ concentration plays a vital role in the metabolism of algae (Beardall et al., 1998), such as *Chlamydomonas reinhardtii* (Renberg et al., 2010). Among the recent single-cell studies, *C. reinhardtii* transcriptome and metabolome have been studied under different CO_2_ concentrations (Fang et al., 2012). These studies provided important information for understanding elevated CO_2_-reponsive gene functions and metabolism. However, changes at the mRNA and metabolite levels do not always reflect changes at the protein level (Washburn et al., 2003; Deyholos, 2010). Thus, studying the protein level changes in response to elevated CO_2_ is important.

Proteomics technologies allow a systemic overview of the cellular physiology in a holistic manner to underscore the underlying metabolic and regulatory mechanisms (Zhang et al., 2012). Oxidative modification of redox-sensitive cysteine (Cys) and methionine (Met) residues in proteins may constitute one of the mechanisms that modulate plant responses to oxidative stress (Navrot et al., 2011). Although multiplex technologies, such as ICAT (Sethuraman et al., 2004), OxICAT (Leichert et al., 2008), cysTMT (cysteine tandem mass tags) (Parker et al., 2012), cysTRAQ (Held and Gibson, 2012), and OxMRM (Wu et al., 2006; Held and Gibson, 2012) were used in redox proteomics, they did not monitor protein level changes in addition to oxidative post-translational modifications (OPTM). Overlooking protein level changes can lead to misleading results in determining redox responsive proteins.

In this study, we employed a double labeling strategy (iTRAQ and iodoTMT) termed iodoTMTRAQ in one experiment, which can provide information on both OPTM and total protein level changes. IodoTMT tags (m/z 126, 127, 128, 129, 130, and 131) were used to label protein thiols responsive to the bicarbonate treatment, and iTRAQ tags (m/z 113, 114, 115, 116, 117, and 118) were used to label the N termini of peptides for analysis of protein-level changes. A total of 47 redox-regulated proteins were significantly changed in response to the bicarbonate treatment (*p* < 0.05). The proteins function in nutrient transport, stress and defense, cell structure modulation, carbohydrate and energy metabolism, protein turnover and fate. These results not only revealed a list of redox responsive proteins and processes, but also suggested an important and interesting redox mechanism in plant cell elevated CO_2_ responses. This redox proteomics approach can be applied to many other areas of plant biology, including abiotic and biotic stress studies.

## Experimental procedures

### *A. thaliana* cell culture and 3 mM NaHCO_3_ treatment

*A. thaliana* (var. Landsberg erecta (LER) suspension cells, kindly gifted by Dr. Joshua L. Heazlewood, University of Western Australia, Australia) were maintained and sub-cultured according to a previous method (Misra et al., 2016). Briefly, the cell culture was grown in 250-ml flasks under light at 130 μmol m^−2^s^−1^ on an orbital shaker at 120 rpm, 22°C, and was sub-cultured weekly. For the control and 3 mM sodium bicarbonate treatment, a 50 mM MES buffer (pH 5.8) buffered MS medium was used. In addition, the buffered MS media were sonicated to remove atmospheric gases before the bicarbonate treatment. The concentrations of bicarbonate and free CO_2_ were 2.34 mM and 0.66 mM, respectively, based on the Henderson–Hasselbalch equation (pH = pK_1_ + log [${\text{HCO}}_{3}^{-}$]/[CO_2_]) (Xue et al., 2011). A value of pK_a_ = 6.352 was used for the calculation. For control experiments, 3 mM NaNO_3_ (a macronutrient in culture media) was added to nullify the effect of excess Na^+^. After 5, 15, 30, 60, and 120 min, both the NaHCO_3_-treated and the control cells were harvested by vacuum filtration and immediately frozen in liquid nitrogen for further analyses. Three independent replicates were prepared for each sample.

### Malondialdehyde (MDA), ROS scavenging enzymes and reductant assay

To detect lipid peroxidation and membrane integrity, MDA content was determined by a thiobarbituric acid (TBA) reaction method (Heath and Packer, 1968). To evaluate the levels of ROS in the suspension cells, H_2_O_2_ content and ${\text{O}}_{2}^{-}$ generation rate were measured. Cells were ground with 0.1% trichloroacetic acid. The homogenate was centrifugation at 15,000 g for 15 min at 4°C and the supernatant was collected for ROS measurement. H_2_O_2_ content was determined spectrophotometrically after reaction with potassium iodide (Ibrahim and Jaafar, 2012), and ${\text{O}}_{2}^{-}$ generation rate was measured using a hydroxylamine oxidization method (Li and Gong, 2005).

For antioxidant enzyme activity analysis, suspension cells were ground to a fine powder in liquid nitrogen and suspended in 50 mM phosphate buffer (pH 7.8). After centrifuged at 15,000 g for 20 min at 4°C, the supernatant was collected for enzyme activity assays. The activities of superoxide dismutase (SOD), catalase (CAT), peroxidase (POD), ascorbate peroxidase (APX), monodehydroascorbate reductase (MDHAR), dehydroascorbate reductase (DHAR), glutathione reductase (GR), glutathione peroxidase (GPX), and glutathione S-transferase (GST) were analyzed as previously described (Suo et al., 2015).

The concentrations of reduced ascorbate (AsA), oxidized ascorbate (dehydroascorbate, DHA) and total ascorbate (AsA+DHA) were determined as previously described (Law et al., 1983). Briefly, 2 g fresh samples were homogenized using a chilled mortar and pestle in 4 mL 5% (w/v) m-phosphoric acid. Following centrifugation at 15,000 g for 15 min at 4°C, the supernatant was used for the analysis of AsA+DHA as well as AsA. The DHA concentration was determined by the difference between total ascorbate and AsA. The concentrations of GSH, GSSG, and total glutathione (GSH+GSSG) were estimated in the supernatants spectrophotometrically as previously described (Griffith, 1980).

### Protein extraction

The harvested cells were grounded in liquid nitrogen. Approximately 300 mg fine powder was transferred into a 2-mL Eppendorf tube and mixed with 1 mL protein extraction buffer 100 mM Tris-HCl (pH 8.8), 10 mM EDTA, 1 M sucrose, 1 mM PMSF and 20 mM N-ethylmaleimide (NEM) for 1 h at room temperature (RT). The NEM in the extraction buffer is to block free thiols and prevent them from further oxidation. The homogenates were centrifuged at 15,000 g for 15 min at 4°C, and the supernatants were added to 5 volume of 100 mM ammonium acetate/methanol. Samples were kept at −20°C overnight and then centrifuged at 15,000 g for 15 min at 4°C. The resulting pellets were washed with 100 mM ammonium acetate/methanol twice, cold 80% acetone twice, and 100% acetone once. The pellets were dissolved in 6 M urea, 1 mM EDTA, 50 mM Tris-HCl (pH 8.5) and 1% SDS. Protein samples were prepared from three independent biological replicates, and protein concentration was determined as previously described (Parker et al., 2012).

### iodoTMT labeling and trypsin digestion

Reverse labeling of thiols were performed as described by Parker et al. (2012). Reduced thiols for reverse labeling were generated by incubation with 5 mM tris (2-carboxyethyl) phosphine for 1 h at 50°C. We labeled 5, 30, and 120 min control samples with 126, 128, and 130 m/z, and the treatment samples with 127, 129, and 131 m/z, respectively. Labeling was performed at 37°C for 2 h in the dark, then quenched with 0.5 M DTT for 15 min at 37°C in the dark. The samples were then separated in a 12% SDS-PAGE gel at 120 v for 5 min and 80 v for 10 min. This step is to clean up the protein samples for in-gel digestion with trypsin (Promega, Madison, WI) at 37°C for 16 h according to the iTRAQ manual (AB Sciex Inc.). Peptides were cleaned up with C_18_ desalting columns (The Nest Group Inc., Southborough, MA) and lyophilized to dryness.

### iTRAQ labeling, strong cation exchange fractionation, and LC-MS/MS

The purified peptides were labeled with iTRAQ reagents according to the manufacturer's protocol (AB Sciex Inc., Framingham, MA, USA). The three time-point control samples were labeled with reporter tags 113, 115, and 117, respectively, and three time-point NaHCO_3_-treated samples were labeled with reporter tags 114, 116, and 118, respectively. The labeling reactions were carried out at 37°C for 2 h. Labeled peptides were desalted with C_18_-solid phase extraction and dissolved in strong cation exchange (SCX) solvent A [25% (v/v) acetonitrile, 10 mM ammonium formate, and 0.1% (v/v) formic acid, pH 2.8]. The peptides were fractionated using an Agilent high performance liquid chromatograph (HPLC) 1260 with a SCX column (PolySULFOETHYL A, 100 × 2.1 mm, 5 μM, 300 Å). Peptides were eluted with a linear gradient of 0–20% solvent B (25% (v/v) acetonitrile and 500 mM ammonium formate, pH 6.8) over 80 min, followed by ramping up to 100% solvent B in 5 min. Peptide absorbance at 280 nm was monitored, and 20 fractions were collected, followed by desalting and lyophilization (Parker et al., 2015).

The fractionated peptides were resuspended in solvent A (0.1% formic acid) and separated on an EASY-nLC1000 system coupled to a Q-Exactive Orbitrap Plus™ mass spectrometer (Thermo Fisher Scientific, Bremen, Germany). The peptides were loaded onto an Acclaim PepMap 100 pre-column (20 mm × 75 μm; 3 μm-C_18_), then separated on a PepMap RSLC EASY-Spray analytical column (250 mm × 75 μm; 2 μm-C_18_) at a flow rate of 350 nL/min using solvent A and B (0.1% formic acid, 99.9% acetonitrile). The solvent gradient was 2–30% solvent B in 100 min, 30–98% solvent B in 10 min, then isocratic of 98% solvent B for 10 min. The separated labeled peptides were analyzed on the Q Exactive Plus™ MS (Thermo Fisher Scientific, Bremen, Germany), which was run in positive ion mode and data dependent scanning with higher energy collisional dissociation (HCD) as previously described by Jones et al. (2013) with minor modifications. The chromatographic peak width was 4 s and the default charge state was 3. The full MS resolution was 70,000 (at m/z 200) with a scan range of 400–2000 m/z, an AGC target of 1e6 and a maximum injection time (IT) of 100 ms. The parameters for MS/MS acquisition were set as follows: 17,500 for MS/MS acquisition, with 105 as the fixed first mass (to accommodate the lower m/z iTRAQ reporter ions), 1e6 for AGC target, 55 ms for maximum IT, 20 for loop count, 2 m/z for isolation window, and 35 for the NCE. The underfill ratio was 1% and the charge exclusion was 1, 6–8, and >8. A lock mass of polysiloxane ion (445.12,003 m/z) was used for real-time mass adjustment.

### Database searching and data analysis

Proteome Discoverer 1.4 (Thermo Fisher Scientific, Bremen, Germany) was used for protein identification based on searching the raw data against the uniprot *A. thaliana* database (52,461 entries downloaded in April, 2015). Proteome Discoverer nodes for spectrum grouper and spectrum selector were set to default with the spectrum properties filter set to a minimum and maximum precursor mass of 300 Da and 5 kDa, respectively. The Sequest HT algorithm was used for protein identification. Parameters were set to two maximum missed cleavage sites of trypsin digestion. Tolerances were set to a 10 ppm precursor mass tolerance and a 0.02 Da fragment mass tolerance. Dynamic modifications included phosphorylation (+79.966 (S, T, Y)), oxidation (+15.995 Da (M)), N-ethylmaleimide (+124.048 Da (C)), iTRAQ8plex (+304.205 Da (N-terminus and K), and iodoTMT6plex (+329.227 Da (C)). Percolator was used for protein identification with parameters of a strict target false discovery rate of 0.01 and a relaxed target false discovery rate of 0.05. Quantification was performed using iodoTMT and iTRAQ reporter ion peak intensities.

Unique peptides were selected for the relative protein quantification. Unique peptide peak intensities for each iodoTMT tag were exported, and ratios were calculated accordingly from the median-normalized peak intensity values. Student's *t*-test (two-tailed) on the log2-transformed treated/control ratios was performed. A peptide with a *p* < 0.05 was considered to be statistically significant. For the iTRAQ data, unique peptides were normalized to the total summation of their intensities based on the 113 tagged peptides. Protein grouping was performed by summation of repeated peptides followed by median calculation for the summed peptides belonging to the same protein. After ratio calculation between each treatment protein value and the corresponding control value, a second normalization step for all the ratios to a median equal to one was carried out. Log transformation of the normalized ratios and t-testing for significant differences between the control and treatment ratios were conducted with the null hypothesis of log2 (averaged ratio) = 0. The significant peptides labeled with iodoTMT were compared and contrasted with the significant proteins quantified via iTRAQ. Student's *t*-test was conducted between the fold change of iodoTMT labeled peptides and the fold change of the corresponding proteins based on iTRAQ. Due to the inherent ratio compression with the iTRAQ/TMT technologies (Ow et al., 2009; Christoforou and Lilley, 2012; Savitski et al., 2013), we use the statistical test to determine significant peptide/protein changes (*p* < 0.05). The proteomics data were deposited to ProteomeXchange via PRIDE repository (Vizcaíno et al., 2014) with accession number PXD004963.

### Protein classification, subcellular location, and hierarchical clustering

To determine the level of functional categorization, a Tair Go annotation analysis was carried out (https://www.arabidopsis.org/tools/bulk/go/index.jsp). In addition, conservative protein functions were deduced from literature. The protein subcellular location was predicted using five internet tools: (1) YLoc (http://abi.inf.uni-tuebingen.de/Services/YLoc/webloc.cgi), confidence score ≥0.4; (2) LocTree3 (https://rostlab.org/services/loctree3/), expected accuracy ≥80%; (3) ngLOC (http://genome.unmc.edu/ngLOC/index.html), probability ≥80%; (4) TargetP (http://www.cbs.dtu.dk/services/TargetP/), reliability class ≤3; (5) Plant-mPLoc (http://www.csbio.sjtu.edu.cn/bioinf/plant-multi/), no threshold value in Plant-mPLoc. Only the consistent predictions from at least two tools were accepted. For the inconsistent prediction results among five tools, subcellular localization for the corresponding proteins was deduced from literature. The definition of functional category was referred from literature. Log (base 2) transformed treatment/control ratios were used for hierarchical clustering analysis using Cluster 3.0 available on the Internet (http://bonsai.hgc.jp/~mdehoon/software/cluster/software.htm). Using a tree algorithm, these proteins were organized based on similarities in the expression profile. These proteins can be joined by short branches as they are very similar to each other, and by increasingly long branches as their similarity decreases. Java TreeView (http://jtreeview.sourceforge.net/) was used for data visualization.

## Results

### Effect of elevated ${\text{HCO}}_{3}^{-}$/CO_2_ on membrane integrity

**Figure 1:**
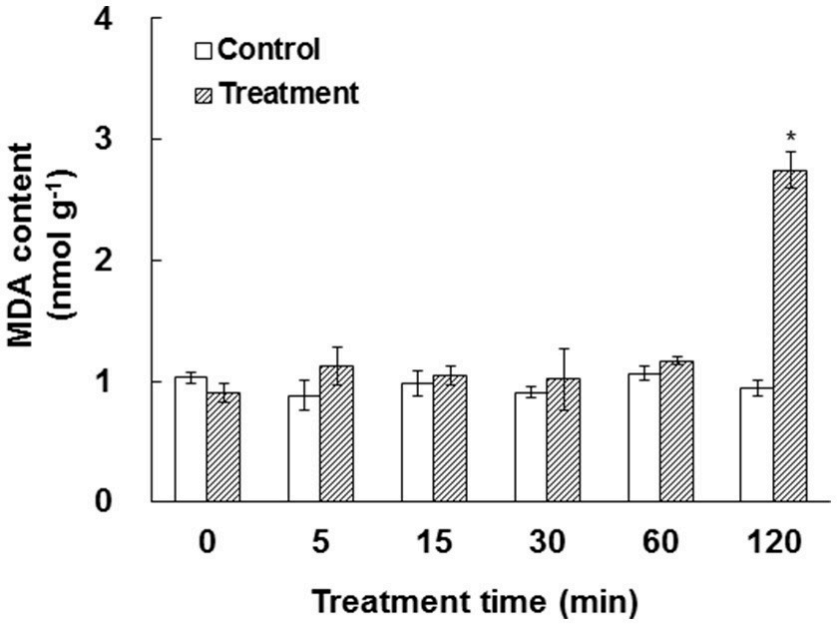
**Effect of elevated ${\text{HCO}}_{3}^{-}$/CO_2_ on malondialdehyde (MDA) in ***A. thaliana*** suspension cells**. The values were determined in control and ${\text{HCO}}_{3}^{-}$/CO_2_ (2.34 mM/0.66 mM) treated samples in a time course of 0, 5, 15, 30, 60, and 120 min. ^*^Indicate significant difference (*p* < 0.05) in the treatment based on Least Significant Difference (LSD) multiple range test. Error bars indicate ± standard error (SE).

In our experiment system, bicarbonate and free CO_2_ were calculated to be 2340 and 660 μM, respectively. Approximately 250 μM bicarbonate was estimated to be in the cytosol of a leaf cell in ambient air (Evans and Von Caemmerer, 1996). Previous work has shown that elevated CO_2_ caused ROS production, which may lead to membrane damage (Ghezzi and Bonetto, 2003; Kolla et al., 2007; Geng et al., 2016). To test the impact of elevated ${\text{HCO}}_{3}^{-}$/CO_2_ on membrane integrity, the MDA content, a reliable indicator for membrane damage (Miller et al., 2010), was measured. Our data showed that MDA content did not change significantly under elevated ${\text{HCO}}_{3}^{-}$/CO_2_ before 60 min, but dramatically increased at 120 min after the treatment (Figure 1), indicating that the membrane structure was relatively stable until the 120 min time-point.

### Enzymes and substrates in the ROS scavenging system

**Figure 2:**
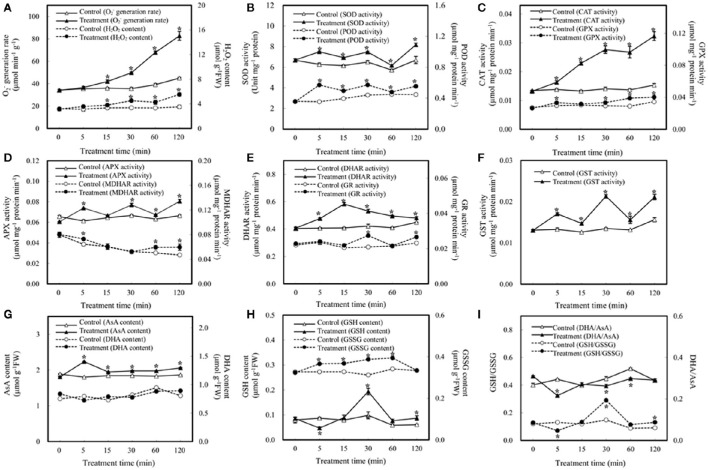
**Effect of elevated ${\text{HCO}}_{3}^{-}$/CO_2_ on the activities of antioxidant-related enzymes and contents of reductants in ***A. thaliana*** suspension cells. (A)** ${\text{O}}_{2}^{-}$ generation rate and H_2_O_2_ content; **(B)** superoxide dismutase (SOD) and peroxidase (POD); **(C)** catalase (CAT) and glutathione peroxidase (GPX); **(D)** ascorbate peroxidase (APX) and monodehydroascorbate reductase (MDHAR); **(E)** dehydroascorbate reductase (DHAR) and glutathione reductase (GR); **(F)** glutathione S-transferase (GST); **(G)** reduced ascorbate (AsA) and oxidized ascorbate (DHA) content; **(H)** reduced glutathione (GSH) and oxidized glutathione (GSSG) content; **(I)** DHA/AsA and GSH/GSSG. Values are means ± SE based on three independent experiments after the cells were treated with elevated ${\text{HCO}}_{3}^{-}$/CO_2_ for 0, 5, 15, 30, 60, and 120 min. ^*^Indicate values that differ significantly between treatment and control at *p* < 0.05 according to LSD multiple range test.

Plants have evolved various strategies to maintain ROS levels, which are regulated by enzymatic and nonenzymatic ROS-producing and-scavenging systems (Apel and Hirt, 2004; Miller et al., 2010). Here we aim to determine the effect of bicarbonate treatment on the ROS scavenging system. As shown in Figure 2A, elevated ${\text{HCO}}_{3}^{-}$/CO_2_ caused dramatic increases in ${\text{O}}_{2}^{-}$ and H_2_O_2_ after 15 min of treatment, indicating potential oxidative stress induced by elevated ${\text{HCO}}_{3}^{-}$/CO_2_. As a key enzyme of scavenging ROS, the activity of SOD was enhanced in the course of elevated ${\text{HCO}}_{3}^{-}$/CO_2_ treatment (Figure 2B). In addition, the activities of various antioxidant enzymes involved in H_2_O_2_ reduction were measured including POD, CAT, four key enzymes involved in ascorbate-glutathione (AsA-GSH) cycle, and GPX. Among them, POD, CAT, and GPX were significantly increased under elevated ${\text{HCO}}_{3}^{-}$/CO_2_ (Figures 2B,C). Interestingly, the activities of enzymes in AsA-GSH cycle varied in the course of the treatment (Figures 2D,E). For example, the activity of APX was increased except at 15 min (Figure 2D), and MDHAR was only activated at 5, 60 and 120 min after the treatment (Figure 2D), while DHAR activity was increased at all the time points (Figure 2E). Moreover, GR activity was increased only at 30 and 120 min after the treatment (Figure 2E). Furthermore, GST activity was increased in the course of the treatment (Figure 2F).

In addition to enzyme activity changes, AsA and GSH are key nonenzymatic antioxidants in the cells, which not only act as substrates in the AsA–GSH cycle, but also provide redox buffer in the cells. In this study, the levels of AsA, DHA, GSSG, and GSH were analyzed. The amount of AsA was increased under elevated ${\text{HCO}}_{3}^{-}$/CO_2_, while DHA remains unchanged (Figure 2G). In addition, the amount of GSH was unchanged except decreased at 5 min and increased at 30 min after the treatment, while the GSSG was increased at all the time points except at 120 min (Figure 2H). Maintenance of a reduced glutathione pool (high GSH/GSSG ratio) is crucial for cellular redox homeostasis, since GSH is utilized to regenerate oxidized ascorbate in the glutathione–ascorbate cycle. In this study, the ratio of GSH/GSSG was increased at 30 and 120 min after the treatment, especially at 30 min (Figure 2I). The ratios decreased at 5 min and remain indifferent from control at 15 min and 60 min. The anti-oxidative enzymes and substrates under elevated ${\text{HCO}}_{3}^{-}$/CO_2_ showed differential changes to regulate the cellular redox state in order to protect the cells against the oxidative stress and cellular damage caused by the bicarbonate treatment.

### Identification of redox-responsive cysteines, peptides, and proteins

**Figure 3:**
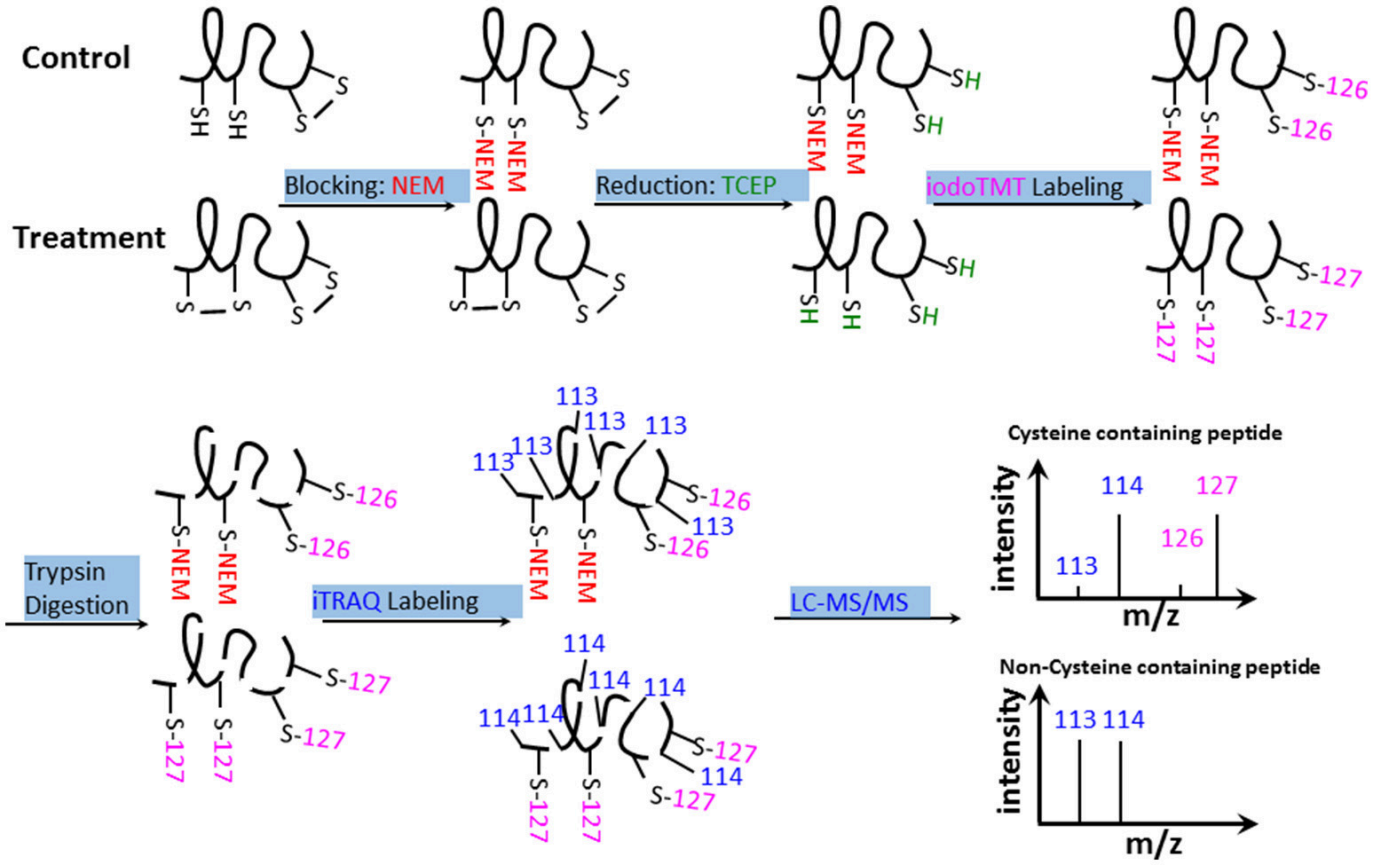
**iodoTMT and iTRAQ-labeling workflow**. Free thiols were first blocked with NEM. Oxidized thiols (e.g., disulfide bonds, sulfenic acids, nitrosylated, or glutathionylated thiols) were reduced with TCEP and labeled with isobaric iodoTMT reagents. The iodoTMT labeled proteins were then digested with trypsin and iTRAQ reagents were used to label the peptides, which were analyzed by LC-MS/MS. Control and ${\text{HCO}}_{3}^{-}$-treated samples were prepared at the same time.

The results of biochemical assays clearly showed cellular redox changes in the course of bicarbonate treatment. To identify proteins that are responsive to the redox changes and may play a role in the bicarbonate responses, we conducted redox proteomic analysis of control and treated samples at 5, 30, and 120 min after the treatment. Proteins were extracted in the presence of NEM, which blocks free thiols to prevent artificial cysteine oxidation during protein extraction (Ghezzi and Bonetto, 2003). After alkylation of the free thiols, oxidized thiols were reduced with TCEP, and the thiols were labeled with iodoTMT tags for quantification. The iodoTMT labeled proteins were then digested with trypsin, followed by iTRAQ labeling of the peptides. Such a reverse-labeling procedure maintains the initial redox state of the proteins and prevents artificial oxidation during sample preparation. Therefore, compared to control samples, the increases of the iodoTMT tags from specific cysteine peptides derived from treated samples indicate the presence of oxidation responsive cysteines and iTRAQ tags indicate the change of total protein level (Figure 3). In this study, a total of 3117 cysteine-containing peptides and 1789 corresponding proteins were observed. Of the cysteine-containing peptides, 903 peptides (29%) were labeled with iodoTMT tags (Tables S1–S3), and 47 cysteine peptides exhibiting significant changes (*p* < 0.05) and present in at least two biological replicates were considered to be redox sensitive (Table S4).

As previously noted, identification of redox proteins may be complicated by protein level changes (Fu et al., 2008; Alvarez et al., 2009). This problem may be resolved by comparing the redox proteomics data with iTRAQ data that show abundance changes for the same proteins (Fu et al., 2008; Alvarez et al., 2009; Zhu et al., 2010, 2014; Tables S1, S2). For example, germination-related protein (GLP, AT3G08030) was identified as a possible redox protein with an iodoTMT intensity change of 0.28-fold at 5 min and 0.43-fold at 30 min after ${\text{HCO}}_{3}^{-}$ treatment (Table 1). However, the iTRAQ data revealed a protein abundance change of 0.76-fold and 0.75-fold, respectively. Thus, the iodoTMT fold change may be attributed to the protein level decrease rather than a cysteine redox change. In contrast, ferredoxin-nitrite reductase was determined to be redox-responsive because it was captured in the iodoTMT labeling without significant protein level changes (Table 1). Here, we have included such proteins as potential redox proteins and excluded those deemed not to be redox-responsive based on comparison with iTRAQ data (Table S5).

**Table 1:** **Significantly changed redox-regulated proteins in response to 3 mM bicarbonate treatment in ***A. thalina*** var. Landsberg erecta**.

**Peptide^a^**	**Protein Accession^b^**	**Protein Description^c^**	**iodoTMT FC^d^**			**iTRAQ FC^e^**			**DiANNA^f^**
			**5**	**30**	**120**	**5**	**30**	**120**	
gQcNAcPSDk	AT1G78850	Curculin-like (Mannose-binding) lectin family protein	0.80	1.24	1.38	0.89	1.10	1.18	Y
lcEcPTVQGVk	AT3G52850	Vacuolar-sorting receptor 1, VSR1	0.77	1.23	0.91	1.00	0.98	1.03	Y
dGIVLNcPHVk	AT3G26370	O-fucosyltransferase family protein, O-FucT-1	0.82	1.38	0.89	1.04	1.03	1.04	Y
sQVQQAVPFLVGcPAcLR	AT4G38350	Patched family protein	0.74	1.21	1.02	0.85	0.98	1.14	Y
aFDMAQcSR	AT4G18030	S-adenosyl-methionine-dependent methyltransferase	0.74	1.08	0.97	0.96	0.96	1.00	Y
gFVGVLHNWcEPFPTYPR	AT1G78240	S-adenosyl-methionine-dependent methyltransferase	0.79	1.12	0.98	1.03	0.84	1.09	Y
vcQVIGAIVDVR	AT5G08670	ATP synthase subunit beta-1, mitochondrial, ATPBM	0.81	1.24	0.89	1.02	0.99	1.00	Y
vLSVLNEATck	AT4G24620	Phosphoglucose isomerase 1, PGI 1	0.88	1.38	0.87	1.04	0.99	1.04	Y
gTcVFPGSAk	AT2G01630	O-Glycosyl hydrolases family 17 protein, GH	0.90	1.44	1.00	1.07	1.07	0.97	N
vPcGYNAPQQVHITQGDVEGk	AT2G16430	Purple acid phosphatase 10, PPA10	0.89	1.15	0.76	1.00	0.91	1.08	Y
gGcYGGMk	AT5G15350	Nodulin-Like Proteins, NLP	0.85	1.57	0.83	0.95	1.03	0.97	Y
aVGScSEcSYTGTFR	AT3G13750	Beta-galactosidase 1, BGAL1	0.56	0.87	1.12	1.03	0.93	1.14	Y
gLVAcTGSQFcGQAIIETk	AT2G15620	Ferredoxin–nitrite reductase, chloroplastic, NR	0.84	1.51	0.87	0.96	0.99	0.99	Y
vcWSTGcFGSDILAAMDR	AT2G05920	Subtilisin serine protease, SSP	0.73	0.89	1.23	0.99	0.92	1.20	Y
hyQDLDFSNVLScAR	AT4G16120	COBRA-like protein 7, COBL7	1.08	1.29	0.96	1.04	0.94	1.02	Y
vDLSMLGTck	AT3G23990	Heat shock protein 60, HSP 60	0.90	1.40	0.74	0.99	1.05	1.00	Y
fAVcLTSGR	AT1G03230	Aspartyl protease-like protein, AP	0.72	1.08	1.17	0.97	0.97	1.11	Y
iLNYVNELcER	AT1G11910	Aspartic proteinase A1, APA1	1.52	0.96	1.17	1.00	1.00	1.00	Y
vGEGPVAQcISGFIALDVAPPR	AT1G11910	Aspartic proteinase A1, APA1	0.35	1.83	1.16	1.00	1.00	1.00	Y
lcVPLVEAQk	AT4G36195	Serine carboxypeptidase S28 family protein, SCP S28	1.03	1.33	0.85	0.96	0.99	1.06	Y
qFNTIPGLMEGTAkPDYATcVk	AT1G15690	Pyrophosphate vacuolar membrane proton pump 1	0.59	0.96	0.80	0.95	0.95	1.06	Y
eGDQcAPQILHVEPNk	AT5G21105	Ascorbate oxidase, Aox	0.95	1.24	0.68	1.13	0.98	1.12	Y
tcAQDEVLR	AT3G08030	Germination-related protein, GLP	0.28	0.43	0.73	**0.76**	**0.75**	0.95	Y
tVcVNQHQVANWNDIcLR	AT5G64816	Uncharacterized protein	0.92	0.86	0.64	1.05	0.85	0.81	Y
tPDVTVDETWFSDPELcEASk	AT5G19440	Alcohol dehydrogenase-like protein, ADH	0.86	0.97	0.73	**0.97**	1.00	1.00	Y
gFHIDGcQASVEAk	AT2G06850	Xyloglucan endotransglucosylase/hydrolase protein 4	0.90	1.20	0.87	0.97	0.92	0.94	Y
gQcNAcPSDk	AT1G78850	Curculin-like (Mannose-binding) lectin family protein	0.77	1.18	1.35	0.89	1.10	1.18	Y
hFEGGDWDQGGTcQR	AT2G14530	Protein trichome birefringence-like 13, TBL13	0.87	1.20	0.91	1.06	0.92	1.07	Y
vHLAGcYIR	AT5G43980	Cysteine-rich repeat secretory protein 56, CRR56	0.93	1.14	0.90	**0.93**	1.00	0.97	Y
sSNQVGSSAcESPER	AT1G19360	Reduced residual arabinose 3, RRA3	0.97	1.17	1.13	0.95	0.96	0.98	N
eAQMcNVLGR	AT5G25100	Transmembrane 9 superfamily member 9, TMN9	0.83	1.06	1.15	0.95	0.97	1.05	Y
eAQMcNILGR	AT5G10840	Transmembrane 8 superfamily member 8, TMN8	0.84	1.09	1.10	0.97	0.94	1.07	Y
vcQVIGAIVDVR	AT5G08670	ATP synthase subunit beta-1, ATPBM	**0.82**	1.19	0.87	1.02	0.99	1.00	N
sLcPSEWVDR	AT1G22450	Cytochrome c oxidase subunit 6b-1, COX6B-1	1.13	1.19	0.81	1.07	1.23	0.81	Y
vVVDTGSELTWVNcR	AT3G12700	Uncharacterized, containing aspartyl protease family	0.82	1.10	1.10	0.99	0.93	1.00	Y
ncAPIMLR	AT4G35000	L-ascorbate peroxidase 3, APX3	**0.92**	1.07	0.97	0.99	1.04	0.99	N
nHcDVAVNSYYQk	AT1G26450	Carbohydrate-binding X8 domain-containing protein	0.86	1.09	0.96	1.01	0.97	0.99	N
gVQGATSHcLGQNFAk	AT3G62120	Proline-tRNA ligase	0.82	1.16	1.07	0.96	0.96	0.98	N
tLNcLPIANIEHFR	AT3G18190	T-complex protein 1 subunit delta, TCPD	1.21	0.93	1.15	0.98	0.94	1.05	Y
eHLcVLk	AT5G20830	Sucrose synthase 1, SUSY1	0.92	1.24	1.10	0.97	0.91	1.03	Y
scSASLAPVILSR	AT3G48990	Oxalate–CoA ligase, 4CLLA	0.98	1.11	0.85	0.98	1.00	0.97	Y
vAVGAPDVLGDcPFSQR	AT1G75270	Glutathione S-transferase, GST	1.08	1.60	0.59	1.00	1.05	0.90	Y
sDDGGADTATDDPcPcA	AT5G20650	Copper transporter 5, COPT5	0.81	0.87	0.99	1.11	0.94	0.90	Y
nAVDmALADSScAGLETTESR	AT1G12230	Transaldolase-like protein, TLP	0.86	1.05	0.77	1.04	1.07	0.89	N
aDWHScLDNR	AT4G35830	Aconitate hydratase 1, ACO1	0.93	0.43	1.03	0.97	0.96	1.02	Y
tTSQDVDESIck	AT4G00100	40S ribosomal protein S13-2, RS13-2	0.99	1.00	1.11	0.98	0.90	0.90	N
rPPLGPGScYAQ	AT3G55260	Beta-hexosaminidase 1, HEXO1	0.99	1.00	1.11	1.00	0.89	1.05	Y

a *The redox-regulated peptides*.

b *Database accession number from Tair10*.

c *The name and abbreviation of the proteins identified*.

d *Fold change between treated and control samples obtained from iodoTMT*.

e *Fold change between treated and control samples obtained from iTRAQ*.

f *Prediction of intra-molecular disulfide bond formation*.

The red and blue numbers indicate

*significantly changed peptides or proteins between treated and control samples (p < 0.05)*.

The sequences of the identified redox-regulated proteins were analyzed for intra-molecular disulfide bond formation using DiANNA software (http://clavius.bc.edu/~clotelab/DiANNA/). Thirty nine of the 47 redox-sensitive peptides were predicted to form intra-molecular disulfide bonds (Table 1). It should be noted that disulfide bonds represent only one possibility of thiol modifications, and other modifications, such as sulfenic acid, S-nitrosylation and glutathionylation (Depuydt et al., 2011) are also reversible modifications. The iodoTMT labeling method will allow identification of these cysteine modifications if specific reductants are used, e.g., ascorbate and copper for specific reduction of S-nitrosylated cysteines (Kovacs and Lindermayr, 2013).

### Gene ontology annotations

**Figure 4:**
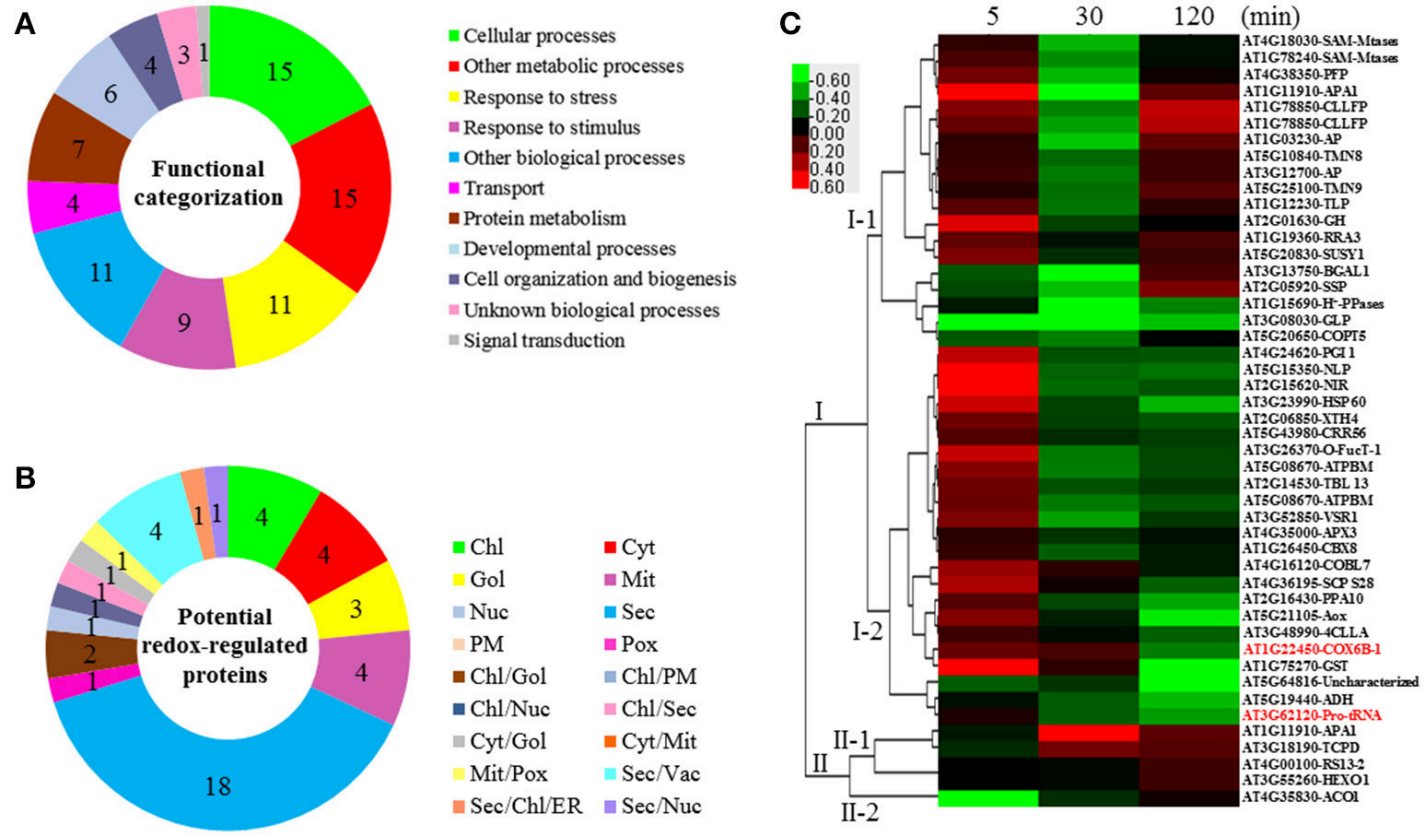
**Functional categorization, subcellular localization, and hierarchical clustering of redox-regulated proteins in ***A. thaliana*** suspension cells under elevated ${\text{HCO}}_{3}^{-}$/CO_2_. (A)** A total of 47 proteins were classified into 11 functional categories on the basis of BLAST alignment, Gene Ontology, and literature. The percentage of proteins in different functional categories is shown in the pie. **(B)** Subcellular localization categories of proteins predicted by internet tools. The numbers of proteins with different locations are shown in the pie. **(C)** Dendrogram of 47 redox-regulated proteins obtained by hierarchical clustering analysis. The columns represent different time point ratios of treatment/control, including 5, 30, and 120 min. The rows represent individual proteins. Two main clusters (I, and II) and subclusters of I and II (I-1, I-2, II-1, and II-2) are showed on the left side. Protein AGI number and name abbreviations are listed on the right side. The increased and decreased proteins are represented in red or green, respectively. The color intensity increases with increasing expression differences, as shown in the scale bar. Chl, chloroplast; Cyt, cytoplasm; ER, endoplasmic reticulum; Gol, Golgi apparatus; Mem, cell membrane; Mit, mitochondria; Nuc, nucleus; PM, Plasma Membrane; Pox, peroxisome; Sec, secreted; Vac, vacuole. Detailed information for protein names can be found in Table 1.

The GO annotation from TAIR was employed to classify the significantly changed proteins and potential redox-regulated proteins. As shown in Figure 4A, analysis of GO terms of relevant biological processes revealed that the redox-regulated proteins were involved in response to stress, transport, protein metabolism, developmental processes, cell organization and biogenesis, as well as response to abiotic/biotic stimulus.

### Subcellular localization and hierarchical clustering

The subcellular localization of the 47 significant redox-regulated proteins was predicted using five internet tools (i.e., YLoc, LocTree3, ngLOC, TargetP, and Plant-mPLoc) and literature information. In total, 18 proteins were predicted to be localized in secreted pathway, four in chloroplast, four in cytoplasm, three in Golgi, four in mitochondria, and one in peroxisome. Besides, 12 proteins were predicted to be localized in two different organelles, and one protein in three organelles (Figure 4B, Table 1, and Table S6).

To understand the protein redox modification dynamics in the time-course experiments, hierarchical clustering analysis of the 47 potential redox proteins revealed two main clusters. Cluster I contained 42 proteins, which were divided into two subclusters. The proteins in subcluster I-1 were mainly involved in transport, photosynthesis, carbohydrate and energy metabolism, and cell structure modulation. Interestingly, these pathways were specially reduced in 30 min elevated ${\text{HCO}}_{3}^{-}$/CO_2_ (Figure 4C). The subcluster I-2 proteins were oxidized at 5 min, but became reduced at either 30 or 120 min. These proteins fell into the categories of secondary cell walls, protein folding and turnover, transport, and ROS scavenging (Figure 4C). Cluster II included five proteins, which were divided into two subclusters. Subcluster II-1 contained proteins oxidized either at 30 or 120 min treatment. Subcluster II-2 had an aconitate hydratase 1 (ACO1) involved in regulating resistance to oxidative stress and cell death, and it was significantly reduced at 5 min elevated ${\text{HCO}}_{3}^{-}$/CO_2_ treatment (Figure 4C).

## Discussion

### An iodoTMTRAQ method for redox protein and total protein profiling

In this study, ROS production was induced by elevated ${\text{HCO}}_{3}^{-}$/CO_2_ (Figure 2A), and ROS can lead to oxidation of sensitive cysteine thiol groups (Go and Jones, 2013). To analyze the protein redox changes, an iodoTMTRAQ method was developed to specifically label cysteine residues and the N-termini of peptides in the same experiment (Figure 3). The reduced cysteines were first blocked using NEM, followed by the reduction of the oxidized cysteines and further labeling of the free thiols with iodoTMT, then digested and iTRAQ were employed to label the peptides for monitoring potential protein level changes. Thus, the increases of the iodoTMT signal from treated samples compared to control samples indicate oxidation of sensitive cysteines (Figure 3). Since the reduced cysteines are blocked prior to the reduction and labeling of the reversibly oxidized cysteine residues, decreases of the iodoTMT signals from treated samples compared to control samples may indicate potentially reduced cysteine residues. Therefore, this methodology can identify proteins that undergo redox changes in addition to mapping of the modified cysteines. Moreover, acquisition of both redox and total protein level information allows for high confident identification of redox proteins and cysteines after normalization against the total protein level changes. Due to the use of the generic reductant TCEP, the iodoTMT labeling method will not differentiate different cysteine redox modifications (e.g., sulfenic acids, disulfide bond, nitrosylation and glutathionylation; Depuydt et al., 2011). If specific reductants are used, e.g., ascorbate and copper for specific reduction of S-nitrosylated cysteines (Kovacs and Lindermayr, 2013), this method will allow identification of different cysteine modifications.

### Redox-sensitive proteins in Arabidopsis cells under elevated ${\text{HCO}}_{3}^{-}$/CO_2_

Carbonic anhydrase can convert CO_2_ into ${\text{HCO}}_{3}^{-}$ for incorporation into cellular metabolism (Tian et al., 2015), and regulate CO_2_ mediated stomatal movement (Hu et al., 2015; Matrosova et al., 2015). It was reported that about 250 μM ${\text{HCO}}_{3}^{-}$ maintained by cytosolic CA activity is present in a leaf cell (Evans and Von Caemmerer, 1996). Thus, the ${\text{HCO}}_{3}^{-}$ concentration we employed for this experiment (2340 μM) is in great excess. Suspension cells are a relatively homogenous group of cells (Misra et al., 2014). Using the suspension cells allows for uniform and concerted cellular reactions in response to elevated ${\text{HCO}}_{3}^{-}$/CO_2_, thereby enhancing the detection of redox-sensitive proteins. In this study, we were able to detect interesting protein redox changes in the processes of ROS scavenging, nutrient/large molecule transport, cell structure modulation, protein folding and assembly, as well as photosynthesis and energy metabolic processes. In the following sections, we discuss these processes in detail.

### ROS homeostasis in response to bicarbonate treatment

**Figure 5:**
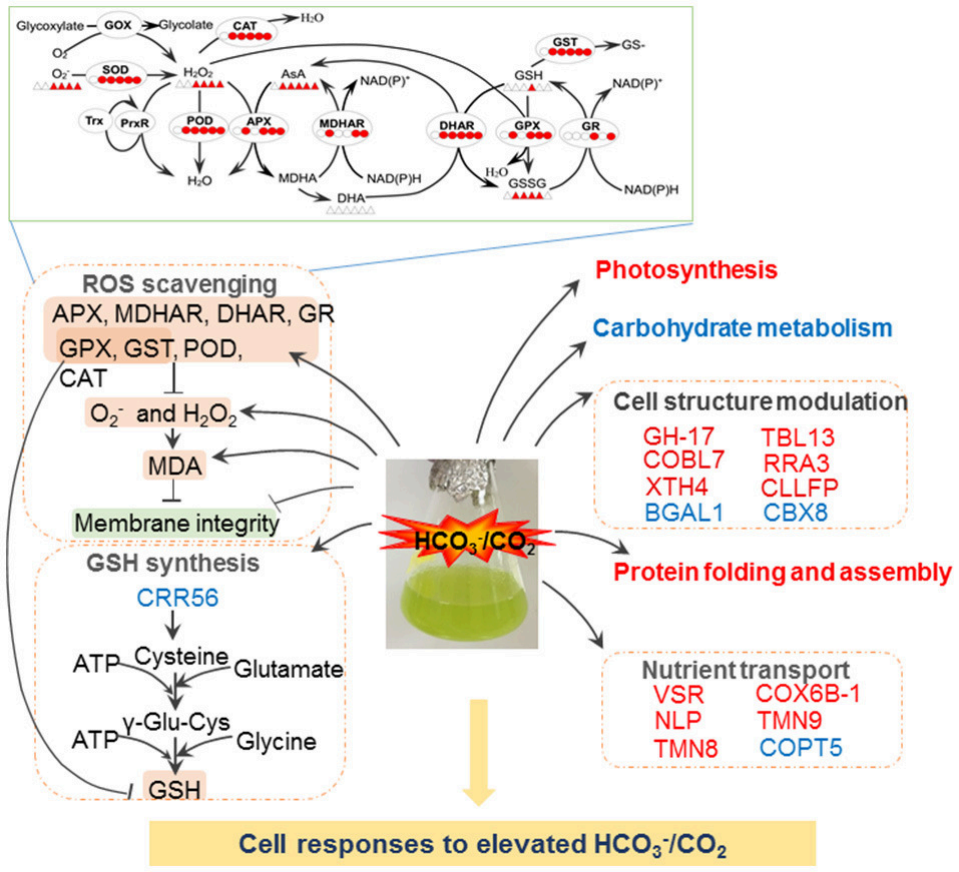
**Schematic presentation of molecular changes in ***A. thaliana*** suspension cells under elevated ${\text{HCO}}_{3}^{-}$/CO_2_**. Elevated ${\text{HCO}}_{3}^{-}$/CO_2_ leads to ROS burst, resulting in the damage to cell membrane. To alleviate ROS toxicity, specific ROS scavenging pathways (top panel) are induced. Elevated ${\text{HCO}}_{3}^{-}$/CO_2_ inactive photosynthesis, protein folding and assemble processes. Importantly, Elevated ${\text{HCO}}_{3}^{-}$/CO_2_ may increase GSH synthesis, and subsequently triggers redox state of redox sensitive proteins involved in cell structure and transport. Solid line with arrow and “T” shape line represent stimulation and inhibition, respectively. The red and green words indicate redox sensitive proteins were oxidized or reduced, respectively. In the ROS scavenging system, the circles/triangles indicate activities/amounts of the enzymes/substrates, respectively, under 0, 5, 15, 30, 60, and 120 min bicarbonate treatment. Abbreviations: APX, ascorbate peroxide; BGAL1, Beta-galactosidase 1; CAT, catalase; CRR56, cysteine-rich repeat secretory protein 56; CBX8, carbohydrate-binding X8 domain-containing protein; CLLFP, curculin-like (Mannose-binding) lectin family protein; COBL7, COBRA-like protein 7; COX6B-1, cytochrome c oxidase subunit 6b-1; COPT5, copper transporter 5; DHAR, dehydroascorbate reductase; H_2_O_2_, hydrogen peroxide; MDA, malondialdehyde; MDHAR, monodehydroascorbate reductase; NLP, nodulin-Like Proteins; GH, glycosyl hydrolases family 17 protein; GOX, glycoxylate oxidase; GPX, glutathione peroxidase; GR, glutathione reductase; GSH, glutathione reduced; GST, glutathione-s-transferases; ${\text{O}}_{2}^{-}$, superoxide anion; POD, peroxidase; ROS, reactive oxygen species; RRA3, reduced residual arabinose 3; SOD, superoxide dismutase; TMN8, transmembrane 8 superfamily member 8; TMN9, transmembrane 9 superfamily member 9; TBL13, protein trichome birefringence-like 13; VSR1, Vacuolar-sorting receptor 1; XTH4, Xyloglucan endotransglucosylase/hydrolase protein 4.

Plants have evolved sophisticated strategies to regulate cellular redox homeostasis and ROS for their growth, development and interaction with the environment (Kültz, 2005). However, dynamic changes of ROS generation and scavenging in response to elevated ${\text{HCO}}_{3}^{-}$/CO_2_ are not clear. The high H_2_O_2_ levels observed in the bicarbonate treated cells compared with control cells (Figure 5) indicate that oxidative stress may have occurred. ROS accumulation activated the antioxidant-related enzymes. For example, SOD showed high activity levels under elevated ${\text{HCO}}_{3}^{-}$/CO_2_ and enabled the dismutation of superoxide into oxygen and H_2_O_2_. To remove excess H_2_O_2_, four key enzymes in the AsA-GSH cycle showed dynamic changes in the course of the treatment (Figure 5, Table 1). The activities of APX, MDHAR, and DHAR were all increased at 5 and 60 min after the treatment, and DHAR activity was also induced at 15 min (Figure 5). The activities of APX, DHAR, and GR were enhanced at 30 min, and those of all the four key enzymes were increased at 120 min (Figure 5). Taken together, the increased activities of the antioxidative enzymes help maintain ROS homeostasis in the response to bicarbonate. Several well-known proteins of the antioxidant system were identified in our study, including APX3, ascorbate oxidase (AOx) and glutathione S-transferase (GST). The cysteine residues of EGDQcAPQILHVEPNK in AOx, NcAPIMLR in APX3, and VAVGAPDVLGDcPFSQR in GST were found to be redox sensitive in this study. The cysteine residue of NcAPIMVR in APX 1 was reported to be redox sensitive in *Arabidopsis* (Liu et al., 2014), and was involved in S-nitrosylation (Fares et al., 2011). These findings suggest that the AsA-GSH cycle may play an important role in bicarbonate responses of Arabidopsis suspension cells.

In addition, GSH and AsA as physiologically relevant antioxidants play a crucial role in plant response to biotic and abiotic stresses (Apel and Hirt, 2004; Hernández et al., 2009). Maintenance of a reduced glutathione pool (high GSH/GSSG ratio; Figure 2I) is crucial for cellular redox homeostasis, since GSH is utilized to reduce DHA and H_2_O_2_ (Foyer and Halliwell, 1976; Leary, 2010). H_2_O_2_ can be reduced to H_2_O by GPX with the consumption of GSH (Mills, 1957), and GST can catalyze the reduction of organic hydroperoxides using GSH as a substrate. The activities of GPX and GST were induced by the bicarbonate treatment, and interestingly, our data showed that GSH amount remained mostly unchanged except for an increase at 30 min after elevated ${\text{HCO}}_{3}^{-}$/CO_2_ treatment (Figure 5). It should be noted that GSH to GSSG ratio was low in the beginning of the treatment (Figure 2I), indicating the suspension cells may have oxidative environment. Nevertheless, many redox responsive cysteine residues and proteins were revealed in this study (Table 1).

### Nutrient and large molecule transport in response to bicarbonate

The roles of nutrient transport have been well-studied under salt and drought stresses (Zhao et al., 2013; Ahmad et al., 2016), but little information is available for elevated ${\text{HCO}}_{3}^{-}$/CO_2_ response. In our redox proteomics results, some nutrient transporters involved in copper, carbohydrate, and protein transport were found to be redox-regulated, such as copper transporter 5 (COPT5), cytochrome c oxidase subunit 6b-1 (COX6B-1), vacuolar-sorting receptor 1 (VSR), pyrophosphate-energized vacuolar membrane proton pump 1 (H^+^-Ppase), transmembrane 8/9 superfamily member 8/9 (TMN8/9), and nodulin-like protein (NLP) (Table 1). Copper delivery is essential for various biological processes, such as antioxidant defense (Hasan and Lutsenko, 2012; Hatori et al., 2012). Copper transport is controlled by GSH through regulating the redox state of copper chaperones (Hatori et al., 2012), which are important in copper sensing and redox homeostasis in *Arabidopsis* (Ohtsu et al., 2001; Attallah et al., 2011). Here we found that two proteins involved in cooper transport, COPT5 and COX6B-1, were redox responsive (Table 1). The functional significance of their redox regulation is intriguing. Another potential redox protein NLP identified in this study (Table 1) was known to be important for transporting nutrients, solutes, amino acids or hormones (Denancé et al., 2014) and was identified in previous studies in *Arabidopsis* (Borner et al., 2002, 2003; Khan et al., 2007). The NLPs are related to phytocyanins, i.e., blue copper proteins that bind a single copper atom and function as electron transporters. Another redox-sensitive protein VSR1 is involved in sorting and targeting aleurain, a vacuolar thiol protease from the trans-Golgi to the lytic vacuoles (Paris et al., 1997). The thiol protease has cysteine residues at the active site, and its activity is known to be regulated by redox (Klomsiri et al., 2011). Redox regulation of the transport-related proteins is an interesting finding.

### Cell structure modulation in response to bicarbonate

Mechanisms in the organization of cytoskeleton and cell wall dynamics have been well studied in the whole plant (Carpita and McCann, 2000). In our redox proteomics data, several cell wall-related enzymes were found responsive to the bicarbonate treatment, e.g., glycosyl hydrolases family 17 protein (GH-17), beta-galactosidase 1 (BGAL1), curculin-like (Mannose-binding) lectin family protein (CLLFP), COBRA-like protein 7 (COBL7), and Xyloglucan endotransglucosylase/hydrolase protein 4 (XTH4). The GH-17, CLLFP, COBL7, and XTH4 were oxidized upon elevated ${\text{HCO}}_{3}^{-}$/CO_2_ treatment for 30 min, and BGAL1 was reduced at 5 min (Table 1). GH-17 hydrolyzes 1,3-beta-glucan polysaccharides found in the cell wall matrix, serving diverse roles in plant defense and development through cell wall remodeling (Free, 2016). BGAL1 is also a member of GHs involved in cell wall metabolism and abscission (Wu and Burns, 2004; Roach et al., 2011). In addition, CLLFP contains mannose-binding sites and a lectin domain (Ferrari et al., 2007). Plant lectins are synthesized via the secretory pathway (Vitale and Chrispeels, 1992; Xiang et al., 2013) and are often found in the inner periphery of the cells in loose association with the cell wall (Kim et al., 2014). COBL2 was found to play a role in the deposition of crystalline cellulose into secondary cell wall structures (Li et al., 2003). However, the role of their redox regulation in response to elevated ${\text{HCO}}_{3}^{-}$/CO_2_ process remains elusive.

### Protein folding and assembly in response to bicarbonate

Our results revealed that proteins involved in synthesis, folding and assembly were redox regulated. Among them, heat shock protein 60 (HSP 60), aspartic protease 1 (ASA1) and 40S ribosomal protein S13-2 (RS13-2) were oxidized in response to elevated ${\text{HCO}}_{3}^{-}$/CO_2_ (Table 1). HSP 60 and RS13-2 may facilitate the correct folding of imported proteins and promote the refolding and proper assembly of unfolded proteins. Notably, ASA1 functions in abscisic acid (ABA) mediated drought response, and overexpression of ASA1 reduced water loss in Arabidopsis (Yao et al., 2012). It is not known whether ASA1 is redox regulated in ABA signaling as some protein kinases (Zhu et al., 2014; Zhang et al., 2016).

### Metabolite and energy adjustment in response to bicarbonate

It is within expectation that photosynthesis proteins were strongly affected by elevated ${\text{HCO}}_{3}^{-}$/CO_2_. Phosphoglucose isomerase 1 (PGI 1) was oxidized after elevated ${\text{HCO}}_{3}^{-}$/CO_2_ treatment for 30 min (Table 1). The studies of *Clarkia xantiana pgi* mutants showed that the plastid PGI controls the rate of starch synthesis and photosynthesis in saturating light and CO_2_ (Kruckberg et al., 1989). Although PGI is not directly required for CO_2_ fixation and operation of the Calvin cycle, its activity is closely related to photosynthesis because photosynthesis cannot continue unless the phosphorylated intermediates are converted into the end product and Pi is recycled (Kruckberg et al., 1989). In addition, a ferredoxin-dependent nitrite reductase (NR) was found to be oxidized at 30 min elevated ${\text{HCO}}_{3}^{-}$/CO_2_ treatment (Table 1). Similar result was also found in *Brassica napus* guard cells (Zhang et al., 2016). NR is present in photosynthetic tissues and cells (Hirasawa et al., 2010). These findings suggest that bicarbonate treatment causes redox changes in photosynthesis and related processes (Lawson et al., 2014). Besides, several redox sensitive proteins involved in carbohydrate metabolism were found to be reduced under elevated ${\text{HCO}}_{3}^{-}$/CO_2_, including oxalate-CoA ligase (4CLLA), carbohydrate-binding X8 domain-containing protein (CBX8), alcohol dehydrogenase-like protein (ADH), and transaldolase-like protein (TLP) (Table 1). The 4CLLA functions in oxalate metabolism. Its reduction may activate the enzyme to promote production of oxalyl-CoA from oxalate, thus decrease the production of H_2_O_2_ from oxalate. In addition, our redox proteomics analysis revealed two ATP synthase subunits were redox sensitive. One ATP synthase subunit beta-1 was reduced at 5 min after elevated ${\text{HCO}}_{3}^{-}$/CO_2_ treatment, and the other one was oxidized at 30 min (Table 1). Interestingly, some ATP synthases in *B. napus* guard cells were also oxidized under ABA treatment (Zhu et al., 2014).

## Conclusions

IodoTMTRAQ was employed to detect redox sensitive proteins and cysteines in bicarbonate treated *Arabidopsis* suspension cells. In this method, redox cysteines were labeled and quantified by the iodoTMT tags and the total protein levels were quantified using iTRAQ tags. We identified 47 potential redox-regulated proteins after considering protein level changes under elevated ${\text{HCO}}_{3}^{-}$/CO_2_ treatment. Many of the proteins, including several stress responsive proteins, were not identified in previous redox proteomics studies. Our results showed the utility of the iodoTMTRAQ method in discovering redox proteins and provided an inventory of bicarbonate responsive redox proteins for future functional studies. The key findings of this study were summarized in Figure 5. Briefly, *A. thaliana* cells can perceive elevated ${\text{HCO}}_{3}^{-}$/CO_2_ and transduce the signal to regulate different cellular processes, including photosynthesis, carbohydrate/energy metabolism, ROS scavenging, cell structure, protein folding and transport. Redox regulation ensures these processes to work cooperatively to achieve a new level of cellular homeostasis under the bicarbonate. In the context of global climate change and continuously rising CO_2_, the results from this study may be used to predict how plants respond and adapt to the environmental changes. Future work should focus on functional characterization of the interesting redox proteins identified in this study, and improve the knowledge toward systemic understanding of the sophisticated and fine-tuned molecular networks underlying plant response to elevated ${\text{HCO}}_{3}^{-}$/CO_2_.

## Author contributions

ZY, KB, and NZ conducted the experiments and drafted the manuscript; SG and TZ advised on experimental design and helped with data analysis; CD acquired the mass spectrometry data, contributed to the data interpretation and edited the manuscript; and SD and SC provided overall guidance and supervision, and finalized the manuscript. All authors approved the final draft of the manuscript.

### Conflict of interest statement

The authors declare that the research was conducted in the absence of any commercial or financial relationships that could be construed as a potential conflict of interest.

## Supplementary material

Table S1 **Protein and peptide level changes determined from the iTRAQ and iodoTMT in replicate 1**. The data were generated using Proteome Discoverer 1.4 by searching the raw MS/MS files against the uniprot *A. thaliana* database.

Table S2 **Protein and peptide level changes determined from the iTRAQ and iodoTMT in replicate 2**. The data were generated using Proteome Discoverer 1.4 by searching the raw MS/MS files against the uniprot *A. thaliana* database.

Table S3 **Protein and peptide level changes determined from the iTRAQ and iodoTMT in replicate 3**. The data were generated using Proteome Discoverer 1.4 by searching the raw MS/MS files against the uniprot *A. thaliana* database.

Table S4 **Peptide level changes determined in at least two replicates of iodoTMT labeling**.

Table S5 **Protein level changes in at least two replicates of iTRAQ labeling**.

Table S6 **Predicted subcellular localization of the differentially changed potential redox-regulated proteins identified in ***A. thalina*** var. Landsberg erecta**.
